# Supplementary material for: Overcoming data challenges through enriched validation and targeted sampling to measure whole-person health in electronic health records
Source: J Biomed Inform. Author manuscript; Available in PMC 2026 Apr 29. (PMC13126318; doi:10.1016/j.jbi.2025.104904)
Supplement: Supp Mat [file NIHMS2164357-supplement-Supp_Mat.pdf]

Supplemental materials for "Overcoming data challenges through enriched validation and targeted sampling to measure whole-person health in electronic health records"

Sarah C. Lotspeich, Sheetal Kedar, Rabeya Tahir, Aidan D. Keleghan, Amelia Miranda, Stephany N. Duda, Michael P. Bancks, Ashish K. Khanna, Joseph Rigdon

August 25, 2025

Original Measurements from Patient Charts

	Cardiovascular System (2 Components)		Metabolic System (3 Components)			Inflammation System (5 Components)					
**Patient ID**	**Systolic Blood Pressure**	**Diastolic Blood Pressure**	**Body Mass Index**	**Triglycerides**	**Cholesterol**	**C-Reactive Protein**	**Hemoglobin A1C**	**Serum Albumin**	**Creatinine Clearance**	**Homocysteine**	**Allostatic Load Index**
1	121.9	73.6	26.1	119.7	26.1	0.5	5.2	3.2	46.3	7.4	
2	134.9	81.5	31.6	173.4	31.6	1.5	8.9	4.1	123.7	11.8	
3	121.5	70.6	47.7	150.3	47.7	6.9	10.9	4.8	156.7	8.5	
4	143.5	96.3	34.5	244.5	265.0	3.0	6.1	4.5	232.0	11.3	

Discretized Measurements for Allostatic Load Index

	Cardiovascular System (2 Components)		Metabolic System (3 Components)			Inflammation System (5 Components)					
**Patient ID**	**Systolic Blood Pressure**	**Diastolic Blood Pressure**	**Body Mass Index**	**Triglycerides**	**Cholesterol**	**C-Reactive Protein**	**Hemoglobin A1C**	**Serum Albumin**	**Creatinine Clearance**	**Homocysteine**	**Allostatic Load Index**
1	<=140	<=90	<=30	<150	<200	<10	<6.5	<3.5	**<110**	<=50	**1**
2	<=140	<=90	**>30**	**>=150**	<200	<10	**>=6.5**	**>=3.5**	>=110	<=50	**4**
3	<=140	<=90	**>30**	**>=150**	**>=200**	<10	**>=6.5**	**>=3.5**	>=110	<=50	**5**
4	**>140**	**>90**	**>30**	**>=150**	**>=200**	<10	<6.5	**>=3.5**	>=110	<=50	**6**

Figure S1: Ten component stressors of the allostatic load index were taken across three body systems (cardiovascular, metabolic, and inflammation), discretized at clinically meaningful thresholds, and combined to create a whole-person health score.

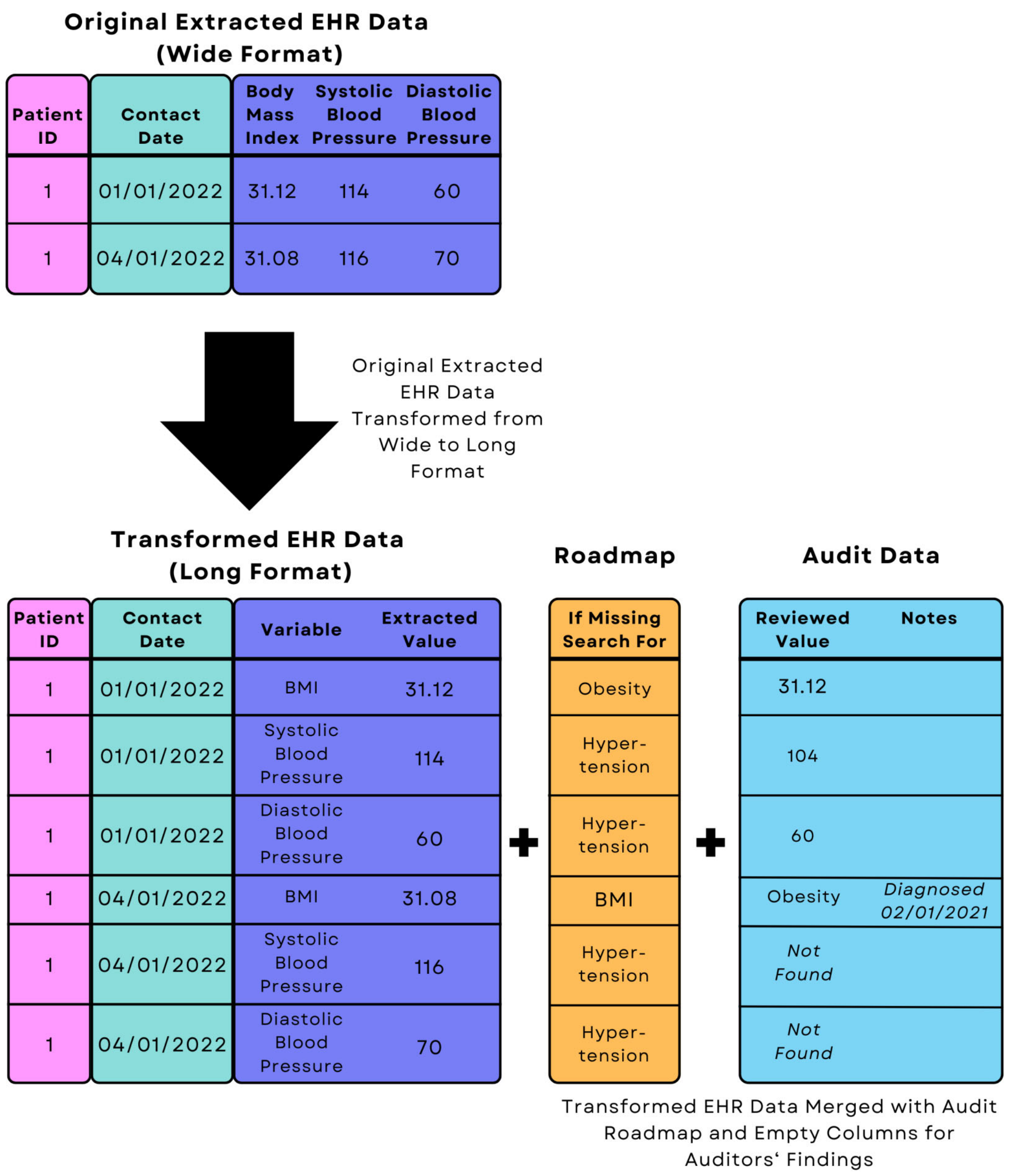

Figure S2: Illustrative example of how the original extracted EHR data for patients' vitals were transformed from a wide format (with one row per patient encounter and columns for different variables) to a long format (with one row per patient encounter per variable), merged with the audit roadmap, and augmented with columns where auditors entered their findings. The same was done with patients' lab data.

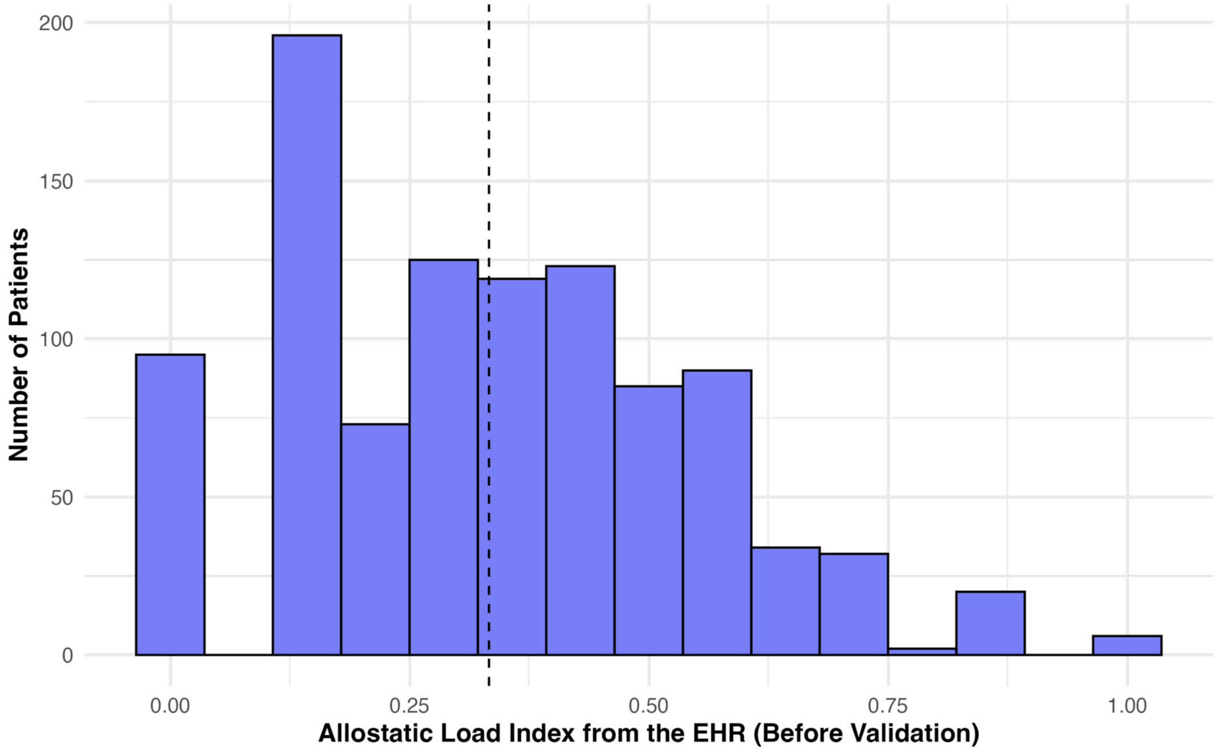

Figure S3: Based on the original extracted electronic health records (EHR) data (before validation), the distribution of the error-prone allostatic load index (ALI) was right-skewed with a median of 0.33 (denoted by the vertical dashed line) and an interquartile range of $(0.17, 0.50)$.

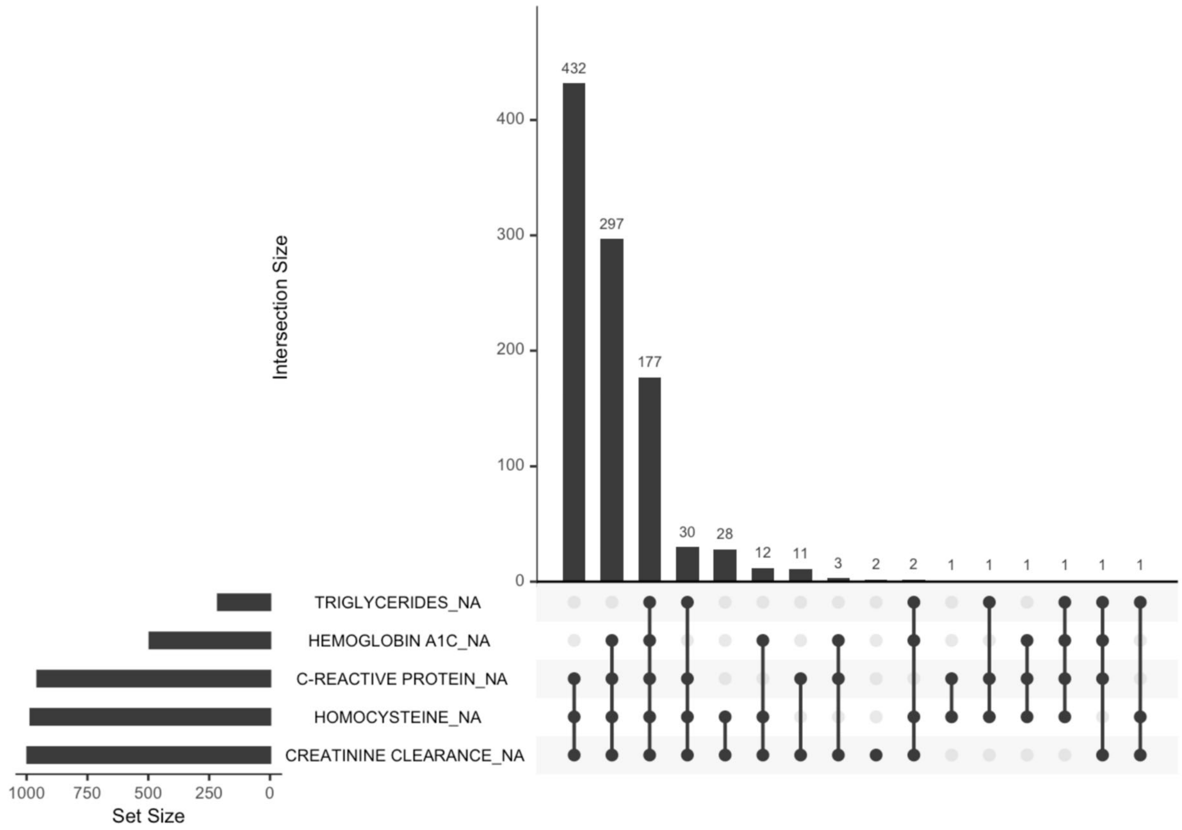

Figure S4: Most common patterns of missing data in the components of the allostatic load index across $N = 1000$ patients in the extracted electronic health records (EHR) data. For example, 432 patients were missing C-reactive protein, homocysteine, and creatinine clearance. This plot was created using the *naniar* package (Tierney et al., 2021).

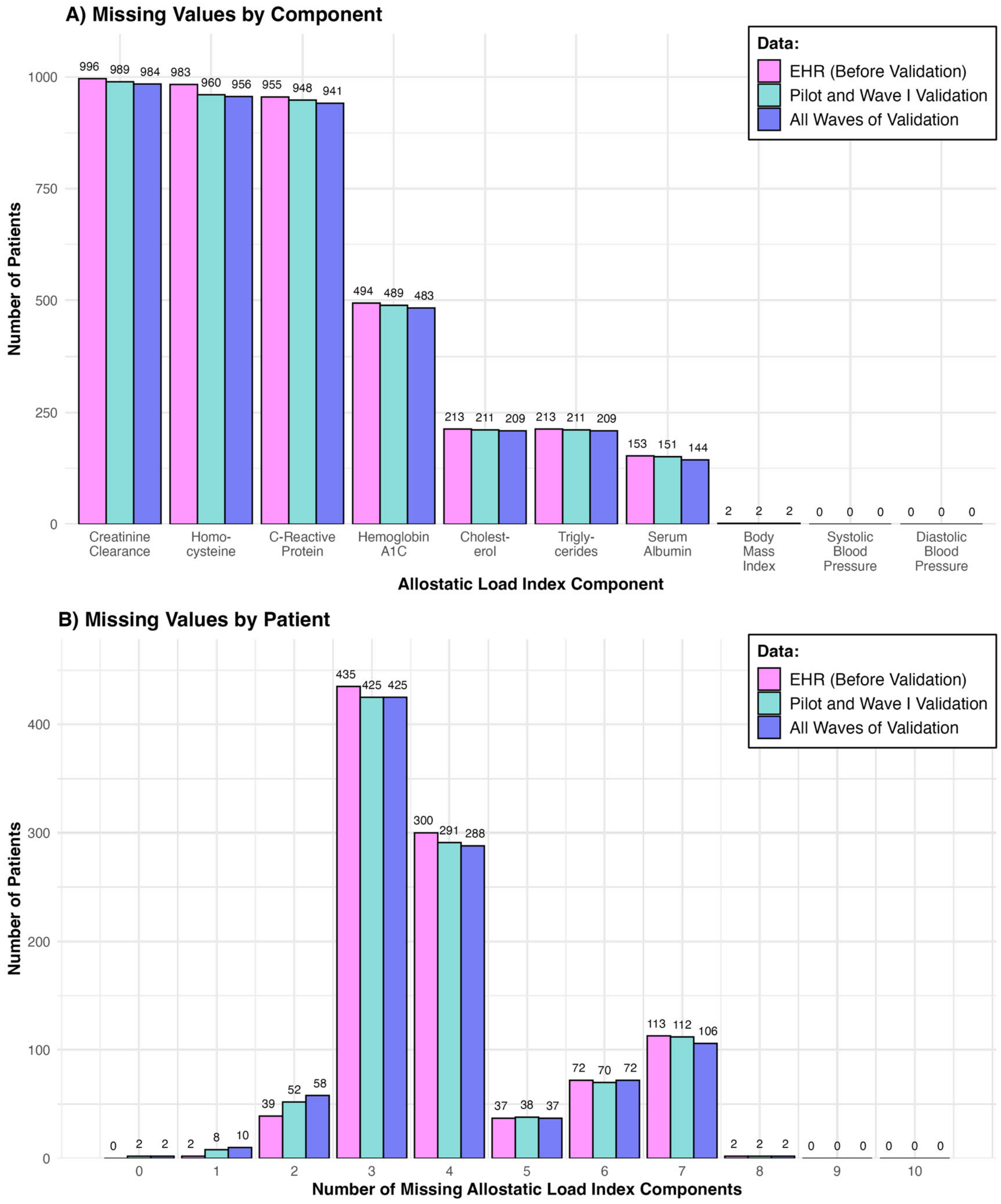

Figure S5: Missingness in the discretized allostatic load index (ALI) components in the extracted electronic health records (EHR) data (before validation), after the Pilot and Wave I validation, and after all waves of validation. In **A)**, missingness is broken down by the ALI component, and in **B)**, it is broken down by the number of missing ALI components per patient.

Component	n	Extracted Value Correct n (%)	Extracted Value Incorrect n (%)	Extracted Value Not Found n (%)	No Auxiliary Information Found n (%)	Auxiliary Information Found n (%)
			Pilot and Wave I Validation			
**Labs (Total)**	**1049**	**854 (81%)**	**4 (< 1%)**	**7 (1%)**	**129 (12%)**	**55 (5%)**
Serum Albumin	385	372 (97%)	3 (1%)	7 (2%)	1 (< 1%)	2 (1%)
Cholesterol	180	175 (97%)	0 (0%)	0 (0%)	3 (2%)	2 (1%)
Triglycerides	180	175 (97%)	0 (0%)	0 (0%)	3 (2%)	2 (1%)
Hemoglobin A1C	142	122 (86%)	1 (1%)	0 (0%)	13 (9%)	6 (4%)
Creatinine Clearance	57	0 (0%)	0 (0%)	0 (0%)	45 (79%)	12 (21%)
C-Reactive Protein	53	7 (13%)	0 (0%)	0 (0%)	38 (72%)	8 (15%)
Homocysteine	52	3 (6%)	0 (0%)	0 (0%)	26 (50%)	23 (44%)
**Vitals (Total)**	**6556**	**3849 (59%)**	**681 (10%)**	**2003 (31%)**	**23 (< 1%)**	**0 (0%)**
Systolic Blood Pressure	2215	1380 (62%)	162 (7%)	663 (30%)	10 (< 1%)	0 (0%)
Diastolic Blood Pressure	2205	1378 (62%)	162 (7%)	655 (30%)	10 (< 1%)	0 (0%)
Body Mass Index	2136	1091 (51%)	357 (17%)	685 (32%)	3 (< 1%)	0 (0%)
			Wave II Validation			
**Labs (Total)**	**718**	**438 (61%)**	**2 (< 1%)**	**12 (2%)**	**195 (27%)**	**71 (10%)**
Serum Albumin	167	134 (80%)	0 (0%)	3 (2%)	10 (6%)	20 (12%)
Cholesterol	124	100 (81%)	0 (0%)	2 (2%)	18 (15%)	4 (3%)
Triglycerides	124	100 (81%)	0 (0%)	2 (2%)	18 (15%)	4 (3%)
Hemoglobin A1C	148	103 (70%)	2 (1%)	4 (3%)	25 (17%)	14 (9%)
Creatinine Clearance	56	0 (0%)	0 (0%)	0 (0%)	42 (75%)	14 (25%)
C-Reactive Protein	51	1 (2%)	0 (0%)	1 (2%)	38 (75%)	11 (22%)
Homocysteine	48	0 (0%)	0 (0%)	0 (0%)	44 (92%)	4 (8%)
**Vitals (Total)**	**3149**	**2052 (65%)**	**141 (4%)**	**956 (30%)**	**0 (0%)**	**0 (0%)**
Systolic Blood Pressure	1055	734 (70%)	13 (1%)	308 (29%)	0 (0%)	0 (0%)
Diastolic Blood Pressure	1057	734 (69%)	12 (1%)	311 (29%)	0 (0%)	0 (0%)
Body Mass Index	1037	584 (56%)	116 (11%)	337 (32%)	0 (0%)	0 (0%)

Table S1: Auditors' findings from 52 patients' data in the *Pilot and Wave I Validation* and 48 patients' data in the *Wave II Validation.* These findings refer to the original numeric lab and vitals measurements (before discretizing them into allostatic load index components), and there could be multiple per patient per variable. The number of validated data points per variable is denoted by n.

**Component**	***n***	**Extracted Value Correct** ***n*** **(%)**	**Extracted Value Incorrect** ***n*** **(%)**	**Extracted Value Not Found** ***n*** **(%)**	**No Auxiliary Information Found** ***n*** **(%)**	**Auxiliary Information Found** ***n*** **(%)**
**Labs (Total)**	**1767**	**1292 (73%)**	**6 (< 1%)**	**19 (1%)**	**324 (18%)**	**126 (7%)**
Serum Albumin	552	506 (92%)	3 (1%)	10 (2%)	11 (2%)	22 (4%)
Cholesterol	304	275 (90%)	0 (0%)	2 (1%)	21 (7%)	6 (2%)
Triglycerides	304	275 (90%)	0 (0%)	2 (1%)	21 (7%)	6 (2%)
Hemoglobin A1C	290	225 (78%)	3 (1%)	4 (1%)	38 (13%)	20 (7%)
Creatinine Clearance	113	0 (0%)	0 (0%)	0 (0%)	87 (77%)	26 (23%)
C-Reactive Protein	104	8 (8%)	0 (0%)	1 (1%)	76 (73%)	19 (18%)
Homocysteine	100	3 (3%)	0 (0%)	0 (0%)	70 (70%)	27 (27%)
**Vitals (Total)**	**9705**	**5901 (61%)**	**822 (8%)**	**2959 (30%)**	**23 (< 1%)**	**0 (0%)**
Systolic Blood Pressure	3270	2114 (65%)	175 (5%)	971 (30%)	10 (< 1%)	0 (0%)
Diastolic Blood Pressure	3262	2112 (65%)	174 (5%)	966 (30%)	10 (< 1%)	0 (0%)
Body Mass Index	3173	1675 (53%)	473 (15%)	1022 (32%)	3 (< 1%)	0 (0%)

Table S2: Combined auditors' findings from 100 patients' data reviewed in any wave of validation (Pilot, Wave I, or Wave II). These findings refer to the original numeric lab and vitals measurements (before discretizing them into allostatic load index components), and there could be multiple per patient per variable. The number of validated data points per variable is denoted by n.

S.1 Additional Details for the SMLEs

S.1.1 Estimating the Log Odds Ratios

Variables Y (indicator of healthcare utilization), X (allostatic load index [ALI]), and Z (age at first encounter) are assumed to be related through the logistic regression model, such that

$$\Pr_{\boldsymbol{\beta}}(Y = y \mid X, Z) = \left[\frac{1}{1 + \exp\{-\mu_{\boldsymbol{\beta}}(X, Z)\}}\right]^{y} \left[\frac{\exp\{-\mu_{\boldsymbol{\beta}}(X, Z)\}}{1 + \exp\{-\mu_{\boldsymbol{\beta}}(X, Z)\}}\right]^{1-y} \tag{S.1}$$

where $y \in \{0, 1\}$ and $\mu_{\boldsymbol{\beta}}(X, Z) = \beta_0 + \beta_1 X + \beta_2 Z$. As mentioned in the main text, we cannot estimate the log odds ratios (logORs) $\boldsymbol{\beta}$ without also estimating the exposure error mechanism relating X to X^* and Z. We chose to do so nonparametrically, allowing the validated exposure X to take on any values and arbitrarily depend on X^* and Z.

Consider the m unique values of X observed in the validation study of n patients ($m \leq n$), denoted by $x_1, \ldots, x_m$. The simplest nonparametric estimator would be discrete probability functions

$$\widehat{\Pr}(X = x_k | X^* = x^*, Z = Z) = \frac{\sum_{i=1}^{N} \mathrm{I}(X_i = x_k, X_i^* = x^*, Z_i = Z)}{\sum_{i=1}^{N} \mathrm{I}(X_i^* = x^*, Z_i = Z)} \tag{S.2}$$

for $k \in \{1, \ldots, m\}$ and $\mathrm{I}(\cdot)$ the usual indicator function. However, in practice, the data often cannot support this estimator due to (i) a small validation study size n, such that a small number of patients will have any particular x_k, and/or (ii) a large number of distinct values x^*, such that a small number of patients will have $X^* = x^*$ and even fewer will further have $X = x_k$. Without even conditioning on additional Z, 34 unique values of X^* are possible. Each patient has between zero and ten non-missing ALI components in the electronic health records (EHR) and has experienced between none and all of them.

In times like these, when the data cannot support the nonparametric estimator in (S.2), smoothing techniques can be used to reduce the number of unique values of (X^*, Z) on which we are conditioning. Following Tao et al. (2017) and Lotspeich et al. (2022), we will use d B-spline "sieves" (Grenander, 1981; Schumaker, 1981) to smooth over (X^*, Z), and

then construct a nonparametric estimator of the exposure error mechanism:

$$\widehat{\Pr}(X = x_k | X^* = x^*, Z = z) = \sum_{k=1}^{m} \mathrm{I}(X_i = x_k) \sum_{j=1}^{d} p_{kj} B_j^q(x^*, z), \tag{S.3}$$

where $B_j^q(x^*, z)$ is the jth B-spline ($j \in \{1, \ldots, d\}$) of order q and p_{kj} is the corresponding coefficient for the kth value of X ($k \in \{1, \ldots, m\}$) and the jth B-spline. For unvalidated patients, it is further assumed that

$$\log\left\{\widehat{\Pr}(X = x_k | X^* = x^*, Z = z)\right\} = \sum_{k=1}^{m} \mathrm{I}(X_i = x_k) \sum_{j=1}^{d} \log(p_{kj}) B_j^q(x^*, z). \tag{S.4}$$

The approximations in (S.3) and (S.4) are then substituted into the observed-data log-likelihood in (2), and we can proceed with estimating the logORs.

The semiparametric sieve maximum likelihood estimators (SMLEs) $\widehat{\boldsymbol{\beta}}$ are obtained via a computationally stable and efficient EM algorithm (Dempster et al., 1977). The covariance matrix for $\hat{\boldsymbol{\beta}}$ is estimated via the profile likelihood approach (Murphy and Van der Vaart, 2000), which numerically approximates derivatives of the observed-data log-likelihood in (2). These SMLEs were proven to have good large-sample statistical properties in Lotspeich et al. (2022). Namely, they are asymptotically consistent and normal and achieve the semiparametric efficiency bound. As the SMLEs proposed here are a special case of Lotspeich et al. (2022), the reader is referred there for the proofs and additional technical details.

S.1.2 Example Code Using the logiSieve Package

Generally, the total sample size N and validation study size n drive the selection of how many B-spline sieves (d) are used for the SMLEs. As the sample sizes increase, we can accommodate more B-spline sieves (i.e., larger values for d), and the data are effectively smoothed less. The order of the B-splines (q) controls the amount of flexibility, and cubic B-splines ($q = 3$) are often a good choice. One could also use k-fold cross-validation to test various choices of q and d. We used cubic B-splines for the preliminary (Pilot and Wave I chart reviews, $n = 52$ validated patients) and final (all waves of chart reviews, $n = 100$

validated patients) SMLEs with $d = 8$ and 16 sieves, respectively, to nonparametrically approximate the exposure error mechanism, as described in Section S.1.

The splines are defined on the original unvalidated ALI variable X^* from the extracted EHR using the *splines* package in R (R Core Team, 2023). We did not think that errors in ALI would depend on patient age, so Z was omitted from the error mechanism. R code to fit this model is available in the *logiSieve* package, which can be downloaded from GitHub at `www.github.com/sarahlotspeich/logiSieve`. An example using this package follows.

If `data` denotes the dataset and column `ALI_STAR` contains the error-prone ALI values X^*, the following code can be used to set up the B-splines using the `bs()` function and then fit the SMLEs using the `logiSieve()` function. For an R script version, see the accompanying GitHub repository at `https://github.com/sarahlotspeich/ALI_EHR`.

```
# Estimate parameters using all audits + the rest of Phase I
## Setup B-splines
library(splines)
B = bs(x = data$ALI_STAR,
       df = 16,
       Boundary.knots = range(data$ALI_STAR),
       intercept = TRUE,
       degree = 3)
colnames(B) = paste0("bs", seq(1, 16))
data = cbind(data, B)
## Fit SMLE model Y ~ X + Z
### RUN ONCE: devtools::install_github("sarahlotspeich/logiSieve", ref = "main")
library(logiSieve)
fit = logiSieve(
+   analysis_formula = ANY_ENCOUNTERS ~ ALI + AGE_AT_ENCOUNTER_10,
+   error_formula = paste("ALI ~", paste(colnames(B), collapse = "+")),
+   data = data)
## View components of the fitted SMLE model
### Table of coefficient estimates for model Y ~ X + Z
```

```
fit$model_coeff
                       coeff         se
Intercept         -1.49015288 0.19551822
ALI                1.13642888 0.37806016
AGE_AT_ENCOUNTER_10 0.09982692 0.05121787
### Covariance matrix for coefficient estimates for model Y ~ X + Z
fit$vcov
            [,1]         [,2]         [,3]
[1,]  0.038227376 -0.042758826 -0.005694905
[2,] -0.042758826  0.142929485 -0.004809294
[3,] -0.005694905 -0.004809294  0.002623271
### Convergence indicator for the coefficient estimates
fit$converged
[1] TRUE
### Convergence message for the coefficient estimates
fit$converged_msg
[1] "Converged"
### Convergence indicator for the variance/standard error estimates
fit$se_converged
[1] TRUE
```

There are additional arguments to the `logiSieve()` function which are often optional. For example, `pert_scale` controls the size of perturbation used in the profile likelihood approximation of the covariance matrix for $\hat{\boldsymbol{\beta}}$. The default is `pert_scale = 1`, such that a perturbation of $n^{-1/2}$ will be used for the numerical derivatives. Sometimes, however, a smaller perturbation may be needed. Alternative values of `pert_scale` may be desired and this choice can be tuned in practice. When fitting the preliminary SMLEs with only $n = 52$ validated patients, we used `pert_scale = 1 / 4`. Since one of the logORs ($\hat{\beta}_2$ on age) was close to zero, the larger default perturbations (with `pert_scale = 1`) were leading to invalid, negative estimates in the covariance matrix, while the smaller perturbation of $0.25 \times n^{-1/2}$ did not.

S.1.3 Predicting Validated Covariates Using the SMLEs

To embed the ALI into the learning health system's EHR, we need to be able to accurately measure it for *all* patients in care. If validated data were available on everyone, we would simply incorporate X into their electronic charts. However, because only a small subset of patients have validated data, we need a reliable, scalable way to predict the more-accurate ALI X across the health system. Fortunately, the SMLEs empower us to do this without requiring any additional model-fitting or analyses.

We can use the definition of conditional expectation and the estimated exposure error mechanism in (S.3) to predict X from X^* and Z as:

$$\begin{aligned}\widehat{X}_i \equiv \widehat{\mathrm{E}}(X_i|X_i^*, Z_i) &= \sum_{k=1}^{m} x_k \widehat{\Pr}(X = x_k|X^* = X_i^*, Z = Z_i) \\ &= \sum_{k=1}^{m} x_k \sum_{j=1}^{d} \hat{p}_{kj} B_j^q(X_i^*, Z_i), \end{aligned} \quad \text{(S.5)}$$

where $\hat{p}_{kj}$ denotes the SMLE of the coefficient for the kth value of X ($k \in \{1, \dots, m\}$) and the jth B-spline ($j \in \{1, \dots, d\}$). The $\hat{p}_{kj}$ are estimated simultaneously with $\hat{\boldsymbol{\beta}}$, so no additional computation is needed to obtain them. When using the `logiSieve()` function, switching the optional `output` argument from `output = "logORs"` (the default) to `output = "all"` will return a vector called `predicted`. This vector contains the predictions $\widehat{X}_i$ for all rows in the dataset. For rows that were validated, the corresponding element in `predicted` will be their actual validated value, i.e., $\widehat{X}_i = X_i$.

```
## Fit SMLE model Y ~ X + Z (with full output)
fit = logiSieve(
+   analysis_formula = ANY_ENCOUNTERS ~ ALI + AGE_AT_ENCOUNTER_10,
+   error_formula = paste("ALI ~", paste(colnames(B), collapse = "+")),
+   data = data,
+   output = "all")
## View components of the fitted SMLE model
### Vector of observed/predicted validated ALI (first 6)
fit$predicted[1:6]
```

```
[1] 0.2222222 0.5714286 0.5555556 0.1666667 0.4000000 0.4285714
```

S.2 Quantifying Inter-Auditor Agreement

At the start of the validation study, we had two auditors on our team. Each of them reviewed the four patients chosen for the Pilot, which allowed us to calculate inter-auditor agreement in this doubly-validated sample. Later, we had two new auditors join the team, and they each reviewed these four patients' records, as well. Ultimately, agreement in the four auditors' findings was calculated using Fleiss' Kappa (Fleiss, 1971), as implemented in the *irr* package in R (Gamer et al., 2019). Variables were found in two tables in the extracted EHR data (vitals and labs), and we looked at the auditors' agreement in each table separately.

Five *vitals* variables were validated: height, weight, body mass index, systolic and diastolic blood pressure. All four auditors reviewed a total of 457 data points across all instances of these vitals variables for two chosen patients. (Due to time constraints, an additional 305 data points for the other two patients were audited by three of the four auditors.) In general, vitals are collected much more often than labs. The Fleiss' Kappa across the auditors ($\kappa = 0.81$) indicated high agreement.

There were six *lab* variables to validate: hemoglobin A1C, serum albumin, total cholesterol, triglycerides, c-reactive protein, and homocysteine. In the interest of time (and having already established agreement with the vitals variables), three of the auditors reviewed all instances of these lab variables for the four chosen patients, leading to a total of 148 triply-audited data points. Here, we observed even higher agreement between the auditors' findings (Fleiss' Kappa $\kappa = 0.90$).

S.3 Additional Detials for the Simulation Studies

S.3.1 Setup and Data Generation

We began by generating complete, error-free data for $N = 1000$ patients based on preliminary estimates from the EHR and the preliminary validation data (Pilot and Wave I) as follows. First, age at first visit (in 10-year increments) Z was simulated from a Poisson distribution with mean = 4.57 (i.e., 45.7 years old). The ten ALI components S_j ($j \in \{1, \ldots, 10\}$) were simulated from Bernoulli distributions with component-specific probability $\Pr(S_j = 1)$ of being "unhealthy." Values for these ten probabilities can be found in the first column of Table S3. From these components, the true ALI value was calculated as $X = \sum_{j=1}^{10} S_j/10$, i.e., the proportion experienced. The indicator of healthcare utilization Y was generated from a Bernoulli distribution with

$$\Pr(Y = 1|X, Z_{shift}) = \left[1 + \exp\left\{-(-1.75 + 1.88X + 0.10Z_{shift})\right\}\right]^{-1},$$

where $Z_{shift} = Z - 1.8$ is a *shifted* version of age that can be interpreted as "years older than 18" (still in 10-year increments). Using Z_{shift} rather than Z in this model was preferable because it makes the intercept interpretable as the expected odds of engaging in the healthcare system ($Y = 1$) for an 18-year-old, rather than a 0-year-old, with an ALI of 0.

Next, we added errors and missingness to the true component data to mimic the versions found in the EHR. The ten error-prone ALI components S_j^* ($j \in \{1, \ldots, 10\}$) were generated from Bernoulli distributions with probabilities dictated by the assumed true positive rate (TPR) = $\Pr(S_j^* = 1|S_j = 1)$ and false positive rate (FPR) = $\Pr(S_j^* = 1|S_j = 0)$. Missingness indicators for the EHR components M_j^* were simulated from Bernoulli distributions with component-specific probability $\Pr(M_j^* = 1)$ of being missing (Table S3). From these two indicators, the error-prone ALI value was calculated as $X^* = \sum_{j=1}^{10}(1 - M_j^*)S_j^*/\sum_{j=1}^{10}(1 - M_j^*)$, i.e., the proportion of *non-missing* components experienced.

Finally, we generated the validation data that were obtained via the chart reviews. Let r denote the assumed data recovery rate ($r \in [0, 1]$), meaning that $(r \times 100)\%$ of the missing

System	Component	Percent "Unhealthy"	Percent Missing
Cardiovascular	Systolic Blood Pressure	0.145	0.000
	Diastolic Blood Pressure	0.058	0.000
Metabolic	Body Mass Index	0.455	0.002
	Triglycerides	0.332	0.213
	Total Cholesterol	0.314	0.213
Inflammation	C-Reactive Protein	0.311	0.955
	Hemoglobin A1C	0.249	0.494
	Serum Albumin	0.987	0.153
	Creatinine Clearance	0.250	0.996
	Homocysteine	0.000	0.983

Table S3: The complete, error-free values for the ten components of the allostatic load index S_j ($j \in \{1, \ldots, 10\}$) were generated from Bernoulli distributions with probability $\Pr(S_j = 1)$ of having an "unhealthy" measurement. Then, missingness in these components was simulated to mimic the amounts in the original electronic health records data.

components were recovered through validation (i.e., auxiliary information from the roadmap was found for them). Equivalently, $(1 - r) \times 100\%$ of the missing components were still missing after validation. Recovery indicators for the ten components R_j ($j \in \{1, \ldots, 10\}$) were independently generated from a Bernoulli distribution with $r = \Pr(R_j = 1)$. Using the recovery indicators R_j and EHR missingness indicators M_j^* together, updated post-validation missingness indicators for the components were defined as

$$M_j^V = \begin{cases} 0 & \text{if } M_j^* = 0 \text{ (i.e., not missing from EHR), and} \\ 1 - R_j & \text{if } M_j^* = 1 \text{ (i.e., missing from EHR).} \end{cases} \tag{S.6}$$

From the second case of (S.6), notice that $M_j^V = 1$ only if $M_j = 1$ and $R_j = 0$ (i.e., the component was missing in the EHR and not recovered through validated). From the true components and updated missingness indicators, the validated ALI was calculated as $X^V = \sum_{j=1}^{10}(1 - M_j^V)S_j / \sum_{j=1}^{10}(1 - M_j^V)$, i.e., the proportion of non-missing components experienced according to the validated data. Notice that $X^V = X$ only if $r = 1$ (i.e., all

missing data could be recovered). R code to replicate these simulations can be found at `https://github.com/sarahlotspeich/ALI_EHR`.

The focus of our simulations was to compare the statistical efficiency in estimating the adjusted logOR on X (controlling for Z) under different validation study designs when we varied (i) the error rates in X^* and (ii) the percent of missing components in X^* that could be recovered for X^V through validation (data recovery rate). We held the total sample size at $N = 1000$ and the validation sample size at $n = 100$, since these were the values in our study. The data generating mechanisms for X, Y, and Z were also fixed, while the error rates and data recovery rates dictated the varied generation of X^* and X^V.

S.3.2 Model Estimation and Validation Study Designs

For all simulations, the first 52 patients were chosen for validation in the Pilot/Wave I via the balanced case-control design from our study (described in Section 2.6.1 of the main text). Then, the following designs were adopted to select the remaining 48 patients for Wave II validation from the remaining 948 that were not already validated. We considered simple random sampling (SRS), case-control sampling (CC) on Y, balanced case-control (BCC) and optimal (OPT) sampling on Y and X^* (discretized at the median), and residual sampling (RS). The OPT design used model estimates from the Pilot and Wave I validation. See Section 2.4 in the main text for further description of these designs.

The *auditDesignR* package, available on GitHub at `www.github.com/sarahlotspeich/auditDesignR`, contains R functions to sample from all five of these designs. Examples using each function are included below. Let `data` denote the simulated dataset, and let `Y`, `Xstar`, `Xval`, and `Z` denote the columns of `data` containing the outcome, error-prone ALI, validated ALI, and age, respectively.

```
# Choose n = 100 patients for validation under different designs
### RUN ONCE: devtools::install_github("sarahlotspeich/auditDesignR", ref =
    "main")
library(auditDesignR)
## Wave I validation: Draw a BCC* of 52 patients from (Y, X*) strata
```

```
### Stratify X* at the median
data$Xstar_strat = as.numeric(data$Xstar <= median(data$Xstar))
### Create Pilot and Wave I validation indicator "V1"
data$V1 = sample_bcc(dat = data,
                   phI = nrow(data),
                   phII = 52,
                   sample_on = c("Y", "Xstar_strat"),
                   wave1_Validated = NULL)
## Wave 2 validation: Draw remaining 48 patients under various designs
### Design 1: Simple Random Sampling (SRS)
data$V2 = sample_srs(phI = nrow(data),
                   phII = 48,
                   wave1_Validated = data$V1 == 1)
### Design 2: Case-Control Sampling (CC)
data$V2 = sample_cc(dat = data,
                  phI = nrow(data),
                  phII = 48,
                  sample_on = "Y",
                  wave1_Validated = data$V1 == 1)
### Design 3: Balanced Case-Control Sampling (BCC)
data$V2 = sample_bcc(dat = data,
                   phI = nrow(data),
                   phII = 48,
                   sample_on = c("Y", "Xstar_strat"),
                   wave1_Validated = data$V1 == 1)
### Design 4: Optimal Stratified Sampling (OPT)
#### Create stratified versions of additional Phase I variables
data$Z_strat = as.numeric(data$Z <= median(data$Z))
data$Xval_strat = as.numeric(data$Xval <= median(data$Xval))
#### Cross-tabulate Phase I strata
stratN = with(data,
```

```
               data.frame(N00 = sum(Y == 0 & Xstar_strat == 0),
                          N01 = sum(Y == 0 & Xstar_strat == 1),
                          N10 = sum(Y == 1 & Xstar_strat == 0),
                          N11 = sum(Y == 1 & Xstar_strat == 1)))
#### Cross-tabulate Wave I Validation strata
wave1_strat = with(data[data$V1 == 1, ],
                   data.frame(n00 = sum(Y == 0 & Xstar_strat == 0),
                              n01 = sum(Y == 0 & Xstar_strat == 1),
                              n10 = sum(Y == 1 & Xstar_strat == 0),
                              n11 = sum(Y == 1 & Xstar_strat == 1)))
#### Fit the MLEs using validated data from Pilot and Wave I
mle_wave1 = twophase_mle(dat = data,
                         Y_val = "Y",
                         Y_unval = NULL,
                         X_val = "Xval_strat",
                         X_unval = "Xstar_strat",
                         addl_covar = "Z_strat",
                         Validated = "V1",
                         nondiff_X_unval = TRUE)
beta_hat = mle_wave1$mod_Y_val$Est[3] ##### Separate logOR on X
eta_hat = with(mle_wave1, ##### And then the rest of the parameters
               c(mod_Y_val$Est[1:2],
                 mod_Y_unval$Est,
                 mod_X_unval$Est,
                 mod_X_val$Est))
#### Create matrix of complete data and estimate the score at the MLEs
complete_data = expand.grid(Y = c(0, 1),
                            Xval_strat = c(0, 1),
                            Xstar_strat = c(0, 1),
                            Z_strat = c(0, 1),
                            V = c(0, 1))
```

```
s_hat = score(comp_dat = complete_data,
            Y_val = "Y",
            Y_unval = NULL,
            X_val = "Xval_strat",
            X_unval = "Xstar_strat",
            addl_covar = "Z_strat",
            Validated = "V",
            beta = beta_hat,
            eta = eta_hat,
            nondiff_X_unval = TRUE)
#### Conduct grid search based to find optimal allocation to the four strata
grid_search = optMLE_grid(phI = nrow(data),
                        phII = 48,
                        phI_strat = stratN,
                        phIIa_strat = wave1_strat,
                        min_n = 0,
                        sample_on = c("Y", "Xstar_strat"),
                        indiv_score = s_hat)
#### Sample remaining patients according to the grid search results
opt_des2 = grid_search$min_var_design
opt_des2[, c("n00", "n01", "n10", "n11")] = opt_des2[, c("n00", "n01", "n10",
   "n11")] - with(wave1_strat, c(n00, n01, n10, n11))
data$V2 = sample_optMLE(dat = data,
                     sample_on = c("Y", "Xstar_strat"),
                     des = opt_des2,
                     wave1_Validated = "V1")
### Design 5: Residual Sampling (RS)
data$V2 = sample_resid(formula = Y ~ Xstar + Z,
                      family = "binomial",
                      dat = data,
                      phI = nrow(data),
```

```
phII = 48,
wave1_Validated = data$V1 == 1)
```

As in Section S.1.2, the SMLE with $d = 16$ cubic B-spline sieves was fit using the `logiSieve()` function in the *logiSieve* package. The errors in X were assumed to be independent of Z, so the B-splines were placed only on X^*. In a small number of replications ($< 4\%$), there were "empty" B-spline sieves (i.e., columns summing to zero) among the validated rows of the data with $d = 16$, which caused errors with the SMLE. This number d was iteratively reduced in these replications until there were no longer empty sieves; most used $d = 15$ or 14, but a few used as small as $d = 8$. Still, the SMLE did not converge within the allowed maximum of 1000 iterations for a very small number of replications ($< 1\%$), which were excluded. All other results are based on 1000 replications.

Each of the following settings was replicated 1000 times for each validation study design. Across replications, we focused on the empirical unbiasedness of the SMLE for the logOR on X (controlling for Z) and the relative efficiency of the SMLE under different targeted validation study designs rather than SRS (an uninformative strategy). Relative efficiency was calculated as the empirical variance of the SMLE under an SRS design divided by the empirical variance of the SMLE under the other designs, with values greater than one indicating efficiency gains over SRS.

S.3.3 Results With Varied Error Rates

Assuming 100% data recovery from the chart review (i.e., $r = 1$), four error settings were considered: (i) *extra low* (TPR = 0.99 and FPR = 0.01), (ii) *low* (TPR = 0.95 and FPR = 0.05), (iii) *moderate* (TPR = 0.8 and FPR = 0.2), and (iv) *heavy* (TPR = 0.5 and FPR = 0.5). These error rates were assumed for each of the ten ALI components, and together they led to correlations of approximately 0.71, 0.63, 0.38, and 0.00 between error-prone X^* and validated X^V, on average.

Under these varied error rates and different validation study designs, the SMLE $\hat{\beta}_1$ for the adjusted log odds ratio on X remained empirically unbiased, i.e., close to the true

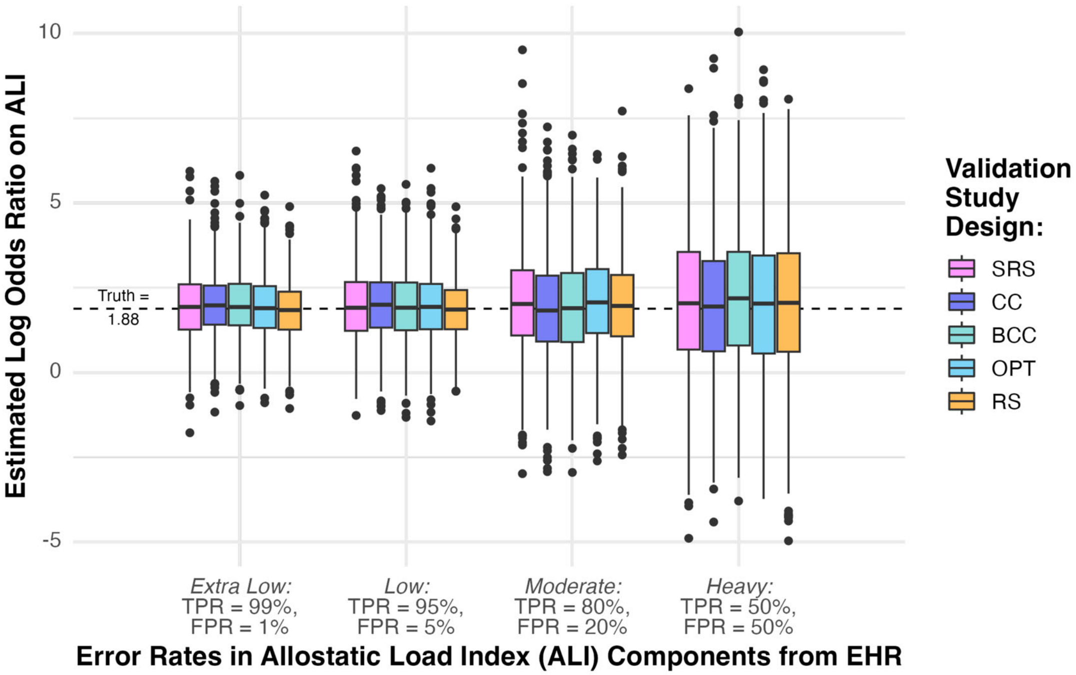

Figure S6: In simulations, the semiparametric sieve maximum likelihood estimator (SMLE) for the adjusted log odds ratio on ALI was empirically unbiased (i.e., close to the truth of $\beta_1 = 1.88$, denoted by the dashed line) under varied error rates and different validation study designs. Five designs were considered: (i) simple random sampling (SRS), (ii) case-control sampling (CC) on Y, (iii) balanced case-control (BCC) and (iv) optimal (OPT) sampling on Y and X^* (discretized at the median), and (v) residual sampling (RS) based on the naive model.

value of $\beta_1 = 1.88$ (Figure S6). Across all settings considered, the SMLE was between $3-9\%$ biased in estimating β_1; this bias would be even smaller if more validated data were available (i.e., larger n). Meanwhile, the naive analysis, which used unvalidated X^* instead of X^V, grew from 48% biased under the extra low error setting to 100% biased under the heavy error setting (data not shown).

Regardless of the validation study design, the SMLE was less efficient (i.e., there was more variability across replications) under worse error rates. This result was expected because the relationship between X^* and X^V gets noisier as the TPR decreases or the

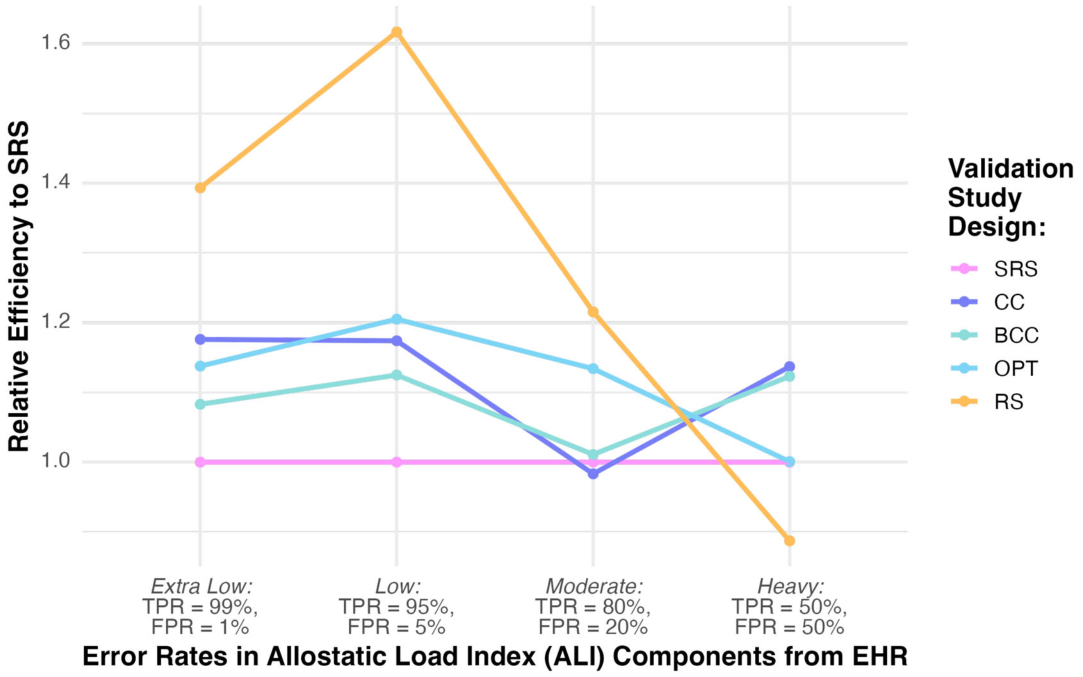

Figure S7: In simulations, the targeted validation study designs offered higher efficiency (i.e., smaller variance) for the semiparametric sieve maximum likelihood estimator (SMLE) log odds ratio estimates on ALI than simple random sampling (SRS) under varied error rates. Four targeted designs were considered: (i) case-control sampling (CC) on Y, (ii) balanced case-control (BCC) and (iii) optimal (OPT) sampling on Y and X^* (discretized at the median), and (iv) residual sampling (RS) based on the naive model.

FPR increases. To a lesser extent, the bias for the SMLE was larger under worse errors, as well, for the same reason.

Under varied error rates, the targeted validation study designs offered higher efficiency (i.e., smaller variance) for the SMLE $\hat{\beta}_1$ than the uninformative SRS strategy (Figure S7). The RS design offered the best efficiency under extra-low, low, and moderate error settings. The other three targeted designs (CC, BCC, and OPT) offered some efficiency gains over SRS, but they were smaller than the gains from the RS design.

In essence, as long as the unvalidated ALI X^* from the EHR data was relatively informative about the validated one X^V, selecting patients with the most extreme residuals

from the naive analysis of Y on (X^*, Z) provided substantially better efficiency than any of the other designs. From the validation data on the first 52 patients (Pilot and Wave I), the correlation between X and X^V was estimated to be even stronger ($R = 0.87$) than in the extra-low error rate considered here ($R \approx 0.71$). The TPR and FPR were estimated to be 100% and < 1%, respectively. Thus, we concluded from these simulations that residual sampling would be the most informative way to select the remaining 48 patients based on our preliminary validation findings on the error severity.

S.3.4 Results With Varied Data Recovery Rates

Assuming 95% true positives and 5% false positives in the EHR components (i.e., TPR = 0.95 and FPR = 0.05), five data recovery rate settings were considered: (i) *full* ($r = 1$), (ii) *high* ($r = 0.9$), (iii) *moderate* ($r = 0.5$), (iv) *low* ($r = 0.25$), and (v) *none* ($r = 0$). As with the errors, these recovery rates were assumed for each of the ten ALI components. The correlation between X and X^V increased as r increased (from $R = 0.74$ to $R = 1$), while the correlation between X^* and X^V instead decreased (from $R = 0.85$ to $R = 0.63$). Essentially, as r increased and more missing components were located, the validated data X^V grew more similar to X and more different from X^*.

The SMLE for the adjusted logOR on X was empirically unbiased under full/high data recovery rates and different validation study designs (Figure S8). While biased for moderate recovery or lower, it was still closer to the truth than the naive estimate was. Even in the most extreme case where the chart reviews recovered 0% of missing ALI components, correcting for errors in the non-missing ones offered estimates that were closer to the truth than the original data were. With a recovery rate of 27% in the preliminary validation data, we expect some residual bias in our SMLEs, regardless of which validation study design we select. Still, our estimates should be closer to the truth than if we had not validated.

Interestingly, under all validation study designs, the SMLE grew more efficient (i.e., less variable) as the data recovery rate decreased. This result was surprising at first since, in general, we expect better efficiency with more correct data. However, it could be explained

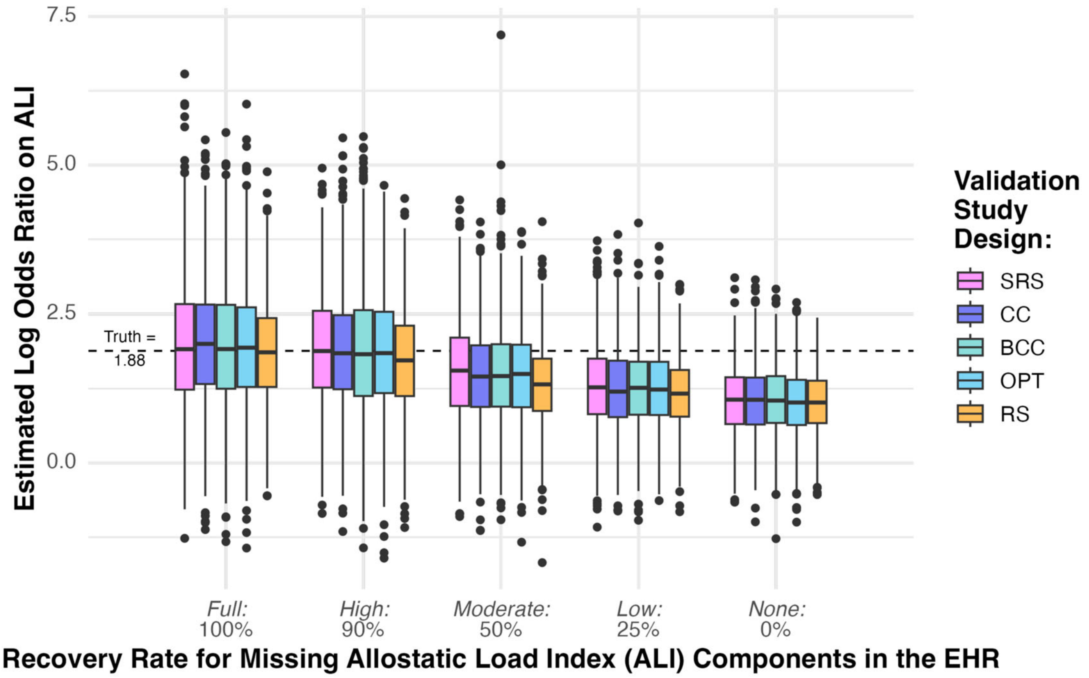

Figure S8: In simulations, the semiparametric sieve maximum likelihood estimator (SMLE) for the adjusted log odds ratio on X was empirically unbiased (i.e., close to the truth of $\beta_1 = 1.88$, denoted by the dashed line) under full/high data recovery rates and different validation study designs. Still, it was closer to the truth for moderate recovery or lower than the naive analysis (i.e., based on the unvalidated EHR data, denoted by the dotted line). Five designs were considered: (i) simple random sampling (SRS), (ii) case-control sampling (CC) on Y, (iii) balanced case-control (BCC) and (iv) optimal (OPT) sampling on Y and X^* (discretized at the median), and (v) residual sampling (RS) based on the naive model.

by the decreasing correlation between X and X^* as r increased. In other words, the error mechanism became noisier and model estimates varied more across replications for higher data recovery rates r.

The RS design offered noticeably higher efficiency for the SMLE than the uninformative SRS or the other targeted designs (CC, BCC, and OPT) regardless of how much missing data was recovered (Figure S9). Having assumed *extra-low* error rates here, we expected RS to be most efficient under 100% recovery at least. Fortunately, the advantages of the RS design appeared consistent across all recovery rates considered. Thus, we once again concluded that residual sampling would be the most informative way to select the remaining 48 patients, now based on our preliminary validation findings on the error severity *and* data recovery rate.

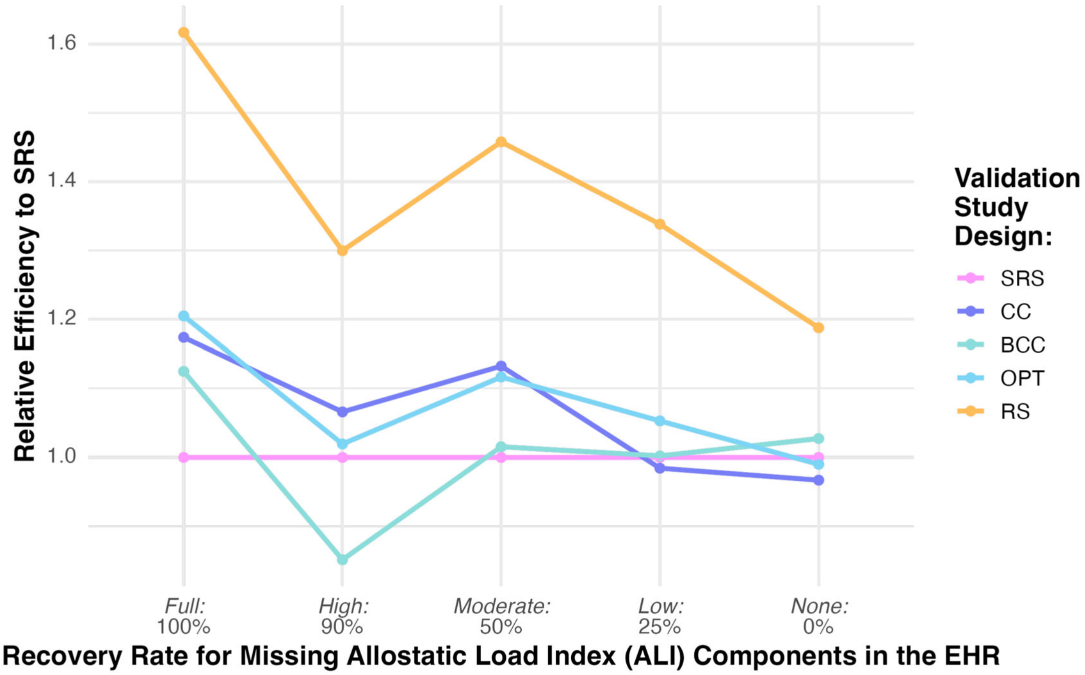

Figure S9: In simulations, the targeted validation study designs offered higher efficiency (i.e., smaller variance) for the semiparametric sieve maximum likelihood estimator (SMLE) log odds ratio estimates than simple random sampling (SRS) under varied data recovery rates. Four targeted designs were considered: (i) case-control sampling (CC) on Y, (ii) balanced case-control (BCC) and (iii) optimal (OPT) sampling on Y and X^* (discretized at the median), and (iv) residual sampling (RS) based on the naive model.

References

Dempster, A. P., N. M. Laird, , and D. B. Rubin (1977). Maximum likelihood from incomplete data via the EM algorithm. *Journal of the Royal Statistical Society: Series B 39*(1), 1–38.

Fleiss, J. L. (1971). Measuring nominal scale agreement among many raters. *Psychological Bulletin 76*, 378–382.

Gamer, M., J. Lemon, I. Fellows, and P. Singh (2019). *irr: Various Coefficients of Interrater Reliability and Agreement.* R package version 0.84.1.

Grenander, U. (1981). *Abstract Inference.* New York: Wiley.

Lotspeich, S., B. Shepherd, G. Amorim, P. Shaw, and R. Tao (2022). Efficient odds ratio estimation under two-phase sampling using error-prone data from a multi-national HIV research cohort. *Biometrics 78*, 1674–1685.

Murphy, S. and A. Van der Vaart (2000). On profile likelihood. *Journal of the American Statistical Association 95*(450), 449–465.

R Core Team (2023). *R: A Language and Environment for Statistical Computing.* Vienna, Austria: R Foundation for Statistical Computing.

Schumaker, L. (1981). *Spline Functions: Basic Theory.* New York: Wiley-Interscience.

Tao, R., D. Zeng, and D. Y. Lin (2017). Efficient semiparametric inference under two-phase sampling, with applications to genetic association studies. *Journal of the American Statistical Association 112*, 1468–1476.

Tierney, N., D. Cook, M. McBain, and C. Fay (2021). *naniar: Data Structures, Summaries, and Visualisations for Missing Data.* R package version 0.6.1.
